# Pattern of arterial inflammation and inflammatory markers in people living with HIV compared with uninfected people

**DOI:** 10.1007/s12350-020-02522-5

**Published:** 2021-02-10

**Authors:** Nevio Taglieri, Rachele Bonfiglioli, Isabella Bon, Pietro Malosso, Andrej Corovic, Matteo Bruno, Elizabeth Le, Bianca Granozzi, Tullio Palmerini, Gabriele Ghetti, Martina Tamburello, Antonio Giulio Bruno, Francesco Saia, Jason M. Tarkin, James H. F. Rudd, Leonardo Calza, Stefano Fanti, Maria Carla Re, Nazzareno Galié

**Affiliations:** 1 Division of Cardiology, Department of Experimental Diagnostic and Specialty Medicine, IRCCS Policlinico di St.Orsola, Alma Mater Studiorum-University of Bologna, Via Massarenti 9, 40138 Bologna, Italy; 2 Division of Nuclear Medicine, Department of Experimental Diagnostic and Specialty Medicine, IRCCS Policlinico di St. Orsola, Alma Mater Studiorum-University of Bologna, Bologna, Italy; 3 Division of Microbiology, Department of Experimental Diagnostic and Specialty Medicine, IRCCS Policlinico di St. Orsola, Alma Mater Studiorum-University of Bologna, Bologna, Italy; 4 Department of Medical and Surgical Sciences, Clinics of Infectious Diseases, IRCCS Policlinico di St. Orsola, Alma Mater Studiorum-University of Bologna, Bologna, Italy; 5 Division of Cardiovascular Medicine, Addenbrookes Hospital, University of Cambridge, Cambridge, UK

**Keywords:** HIV, ^18^F-fluorodeoxyglucose-positron emission tomography, Arterial inflammation

## Abstract

**Study Design:**

To compare arterial inflammation (AI) between people living with HIV (PLWH) and uninfected people as assessed by ^18^F-Fluorodeoxyglucose (^18^F-FDG)-positron emission tomography (PET).

**Methods:**

We prospectively enrolled 20 PLWH and 20 uninfected people with no known cardiovascular disease and at least 3 traditional cardiovascular risk factors. All patients underwent ^18^F-FDG-PET/computed tomography (CT) of the thorax and neck. Biomarkers linked to inflammation and atherosclerosis were also determined. The primary outcome was AI in ascending aorta (AA) measured as mean maximum target-to-background ratio (TBR_max_). The independent relationships between HIV status and both TBR_max_ and biomarkers were evaluated by multivariable linear regression adjusted for body mass index, creatinine, statin therapy, and atherosclerotic cardiovascular 10-year estimated risk (ASCVD).

**Results:**

Unadjusted mean TBR_max_ in AA was slightly higher but not statistically different (*P* = .18) in PLWH (2.07; IQR 1.97, 2.32]) than uninfected people (2.01; IQR 1.85, 2.16]). On multivariable analysis, PLWH had an independent risk of increased mean log-TBR_max_ in AA (coef = 0.12; 95%CI 0.01,0.22; *P* = .032). HIV infection was independently associated with higher values of interleukin-10 (coef = 0.83; 95%CI 0.34, 1.32; *P* = .001), interferon-γ (coef. = 0.90; 95%CI 0.32, 1.47; *P* = .003), and vascular cell adhesion molecule-1 (VCAM-1) (coef. = 0.75; 95%CI: 0.42, 1.08, *P* < .001).

**Conclusions:**

In patients with high cardiovascular risk, HIV status was an independent predictor of increased TBR_max_ in AA. PLWH also had an increased independent risk of IFN-γ, IL-10, and VCAM-1 levels.

**Supplementary Information:**

The online version of this article (10.1007/s12350-020-02522-5) contains supplementary material, which is available to authorized users.

## Introduction

The use of antiretroviral therapy (ART) has dramatically reduced the AIDS-related mortality of people living with HIV (PLWH).^1^ Consequently, PLWH are facing a rising burden of chronic diseases, with cardiovascular disease being a major cause of non-AIDS-related morbidity and mortality.^2^,^3^ Several studies have shown that PLWH have a 1.5- to 2-fold increased risk of myocardial infarction^4^ (MI) and stroke^5^ compared with uninfected people. This excess cardiovascular risk is likely due to an interplay of several mechanisms including traditional risk factors, HIV-related factors such as chronic inflammation and immune activation,^6^ ART-related dyslipidemia,^7^ co-infections,^8^ and disparities in care delivery.^9^,^10^ Accordingly, cardiovascular risk prediction tools, derived from and used in the general population, may underestimate the risk of atherosclerosis-associated cardiovascular events in PLWH.^11^,^12^^18^F-Fluorodeoxyglucose (^18^F-FDG)-positron emission tomography (PET) imaging can report on arterial inflammation associated with atherosclerosis, since glucose is the major substrate for macrophages resident in plaque.^13^,^14^ It has also been shown that ^18^F-FDG uptake in the ascending aorta is associated with future cardiovascular events and provides incremental information above traditional risk factors.^15^ Results from previous studies that have investigated patterns of arterial ^18^F-FDG-PET in PLWH and control subjects have been inconsistent, some studies suggesting an increased arterial inflammation in HIV patients,^16^ others refuting this association.^17^ These studies focused on patients with low cardiovascular risk; however, in clinical practice, many patients with HIV have a high cardiovascular risk based on conventional risk factors.^3^

Therefore, we performed a prospective study of subjects without known cardiovascular disease but with at least 3 traditional risk factors, with the main aim to compare arterial inflammation, as assessed by ^18^F-FDG-PET scan of ascending aorta (AA), descending aorta (DA), and carotid arteries (CAs) between PLWH and uninfected people.

## Methods

### Patients

Between November 2017 and July 2019, PLWH and control subjects were prospectively screened during routine outpatient clinic visits of the Department of Infectious Disease and of the Cardiology Unit at St. Orsola University Hospital of Bologna, respectively. They were then enrolled if they met the following inclusion criteria: (1) at least 3 of the following cardiovascular risk factors: (a) age > 55 years for men or > 65 for women, (b) hypertension, (c) hypercholesterolemia, (d) diabetes mellitus, (e) smoking, and (f) family history of coronary artery disease and (2) release of written consent. Exclusion criteria were as follows: known or suspected cardiovascular disease, acute or chronic infections, systemic inflammatory diseases, corticoid treatment, malignancies, alcoholism, mental illness or drug dependence, and slack of given informed consent. PLWH on ART for less than 6 months or with HCV co-infection were also excluded.

The study was conducted in accordance with the principles of the most recent revision of the Declaration of Helsinki and approved by the Institutional Review Board/Ethics Committee.

Informed consent was obtained from all individual participants included in the study.

### FDG-PET Protocol and Analysis

After receiving a cardiology evaluation, all enrolled patients underwent ^18^F-FDG–PET/computed tomography (CT). To minimize ^18^F-FDG myocardial uptake, patients underwent a low-carbohydrate, high-protein, and high-fat dinner the night before followed by > 12 hours fasting until the scan was performed.^18^^18^F-FDG-PET/CT scanning from the jaw to T12 vertebrae was performed using a Hybrid Scanner (STE, GE-Healthcare). CT images were acquired at 120kV, 80mA, and slice thickness of 3.75mm. PET images were acquired for 10 min/bed position 90 min after the administration of 3.7 MBq/kg ^18^F-FDG and were corrected for attenuation on the basis of the CT data.

Fully anonymized PET/CT images were independently analyzed using Horos^TM^ imaging software (https://horosproject.org) by two external investigators in Cambridge, UK, (AC, EL), who were blinded to the patients’ clinical information. ^18^F-FDG uptake was measured within the wall of the thoracic AA, thoracic DA, and both CAs. Maximum standardized uptake values (SUVs) were calculated on axial plane slice by slice from regions of interest drawn around the vessel wall. The superior vena cava was used as the reference vessel for correction of blood pool activity in the aorta, and the jugular vein was used for the carotids. For each aortic slice, the maximum target-to-background ratio (TBR_max_) was calculated by dividing SUV_max_ of the artery segments with the SUV_mean_ (average of 5 consecutive slices) of the superior vena cava or jugular vein.

Subsequently, TBR_max_ was averaged for each vessel of interest (AA, DA and CAs). This approach (whole vessel method) has been suggested for the assessment of global vascular inflammation as a marker of cardiovascular risk.^19^ As secondary analyses, we also measured the following: (1) the average TBR_max_ in the most diseased segment of the index vessel defined as the arterial slice with the highest ^18^F-FDG uptake, averaged with the slice above and below,^20^ and (2) the average TBR_max_ of active segments with TBR_max_ ≥ 1.6.^21^ Inter- and intraobserver reproducibility of TBR measurements were tested by 2 independent observers using 10% of the aortic and carotid scans (n = 4 for both), selected at random, with 1 week between intraobserver readings.

### Biochemical Measurements

In all patients, 2 venous blood samples (10 mL each) were taken for measurement of biomarkers before undergoing PET evaluation, on the same day. Levels of biomarkers were determined on plasma at the department of Microbiology of our Institution, in a blinded fashion. Supplemental Table 1 shows the full list of biomarkers analyzed, along with the specification of each assay according to the manufacturer. In HIV patients, peripheral blood mononuclear cells were recovered from fresh blood by density gradient centrifugation using Ficoll-Paque Plus (Ficoll-Histopaque, Pharmacia, Uppsala, Sweden). HIV-DNA was quantified using the HIV-1 DNA test (Diatheva , PU, Italy). Plasma levels of HIV-RNA were determined by Roche Cobas AmpliPrep/Cobas TaqMan HIV-1 test version 2.0.

**Table 1:** Baseline characteristics

Variable	HIV+	HIV−	*P* value
No. of patients	n = 20	n = 20	
Age, years, median (25th–75th)	63 (56–70)	65 (57–73)	.53
Male gender—no. (%)	17 (85)	11 (55)	.04
Body mass index, Kg/mq	26 (24–29)	28 (26–32)	.07
Hypercholesterolemia—no. (%)	18 (90)	15 (75)	.02
Hypertension—no. (%)	16 (80)	19 (95)	.15
Smokers—no. (%)	9 (45%)	10 (50%)	.8
Diabetes—no. (%)	3	2	.6
Family history of CAD—no. (%)	4 (21)	6 (30)	.5
Systolic blood pressure, median (25th–75th)	135 (120–155)	138 (130–148)	.87
Diastolic blood pressure, median (25th–75th)	87 (80–90)	80 (80–90)	.13
Laboratory findings			
Creatinine. mg/dL. median (25th–75th)	1.0 (0.9–1.1)	0.81 (0.8–1)	.02
Total cholesterol mg/dL median (25th-75th)	240 (210–258)	204 (189–223)	.006
HDL-C mg/dL median (25th–75th)	52 (43–55)	48 (42–59)	.87
LDL-C mg/dL median (25th–75th)	156 (131–172)	127 (101–159)	.02
ACC_ASCVD equation, median (25th–75th)	16.55 (12.6–31.7)	16.25 (10.0–31.2)	.64
LVEF, %, median (25th–75th)	64 (60–65)	63 (60–65)	.5
Medications			
Anti-hypertensive medication, no. (%)	14 (70)	17 85)	.23
Statins, no. (%)	10 (50)	9 (45)	.75

*ACC/ASCVD*, American College of Cardiology/atherosclerotic cardiovascular disease; *LVEF*, left ventricle ejection fraction

### Statistical Analysis

Mean TBR_max_ in AA was selected as the primary endpoint. Based on previously reported values in HIV population^16^ (mean TBR_max_ = 2.23) and assuming a SD = 0.35,^22^ we estimated that a sample size of 40 subjects would have a 85% study power to detect a 15% between-group difference of mean TBR_max_ in AA.

Continuous and categorical variables are presented as median (interquartile range [IQR]) and frequencies (percentages), respectively. For comparisons between groups, the Mann-Whitney U test was used for continuous variables and Fisher’s exact test was used for categorical variables. We assessed the normality of the distribution of TBR values and biomarker levels both by plotting histograms and with the Shapiro–Wilk test; in case of discrepancies, variables were log-transformed, as a conservative approach.

After log-transformation of variables not normally distributed, the independent relationships between HIV status and either TBR values or biomarkers were evaluated by linear regression using a multivariable model including: American College of Cardiology/atherosclerotic cardiovascular disease (ACC/ASCVD) risk prediction function,^23^ body mass index (BMI), creatinine, and statin therapy. The ACC/ASCVD function includes the following variables: age, gender, race, total cholesterol, HDL, systolic and diastolic blood pressure, medications for hypertension, smoke, and diabetes (Model 1). A second multivariable model (Model 2) was constructed including the following individual variables: gender, body mass index, LDL-cholesterol (not included in the ACC/ASCV function), and creatinine.

Relationships between serum biomarkers and mean TBR_max_ in AA, DA, or CAs were investigated with the use of unadjusted linear regression. Those associations showing a *P* value < .1 were then tested in multivariable linear regression models as above. A two-tailed *P* value < .05 was considered statistically significant. All analyses were performed with STATA 14.0 software (STATA Corporation, College Station, Tex).

## Results

Of 65 patients screened, 40 (20 PLWH and 20 uninfected people) were enrolled (Supplemental Figure 1).

PLWH were more likely to be male and to have a history of hypercholesterolemia. They also had higher values of creatinine, total cholesterol, and LDL-cholesterol. Patients with no HIV had a higher body mass index (Table 1). The majority of PLWH showed well-controlled HIV disease (Supplemental Table 2). The minimum ART duration was 3.2 years.

### Arterial ^18^F-FDG Uptake and HIV Status

The reproducibility of TBR_max_ measurements was good for both intraobserver observations (AA: absolute agreement intraclass coefficient value [ICC] 0.97; 95%CI 0.45,0.99; carotid artery ICC 0.90, 95%CI 0.85-0.94) and interobserver observations (AA ICC:0.98; 95%CI 0.61-0.99; carotid artery ICC 0.95; 95% CI 0.92-0.97).

**Figure 1:**
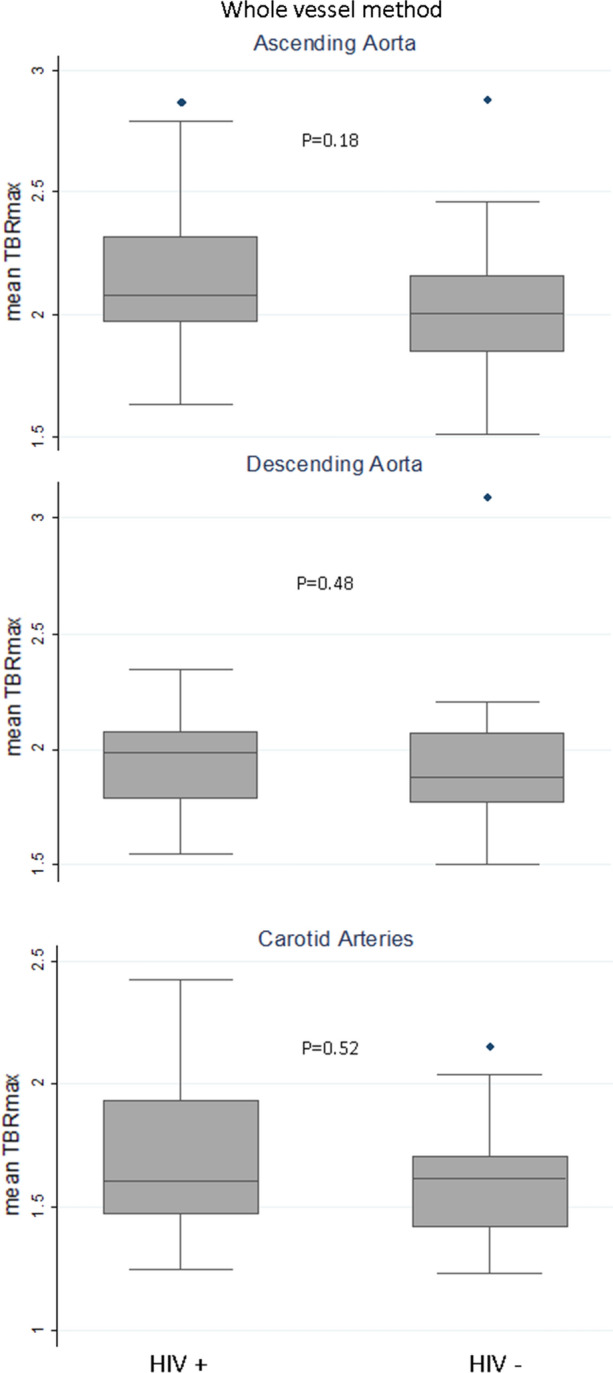
Arterial mean TBRmax by whole vessel method. Box plot (median and 25th–75th percentiles) for median value of mean TBRmax according the HIV status. *TBR*, target-to-background ratio

The distribution of the primary endpoint (mean TBR_max_ in AA) resembled normal distribution, however, showing a discrepancy with the Shapiro–Wilk test for normality (*P* = .019). Figure 1 shows that the median value of mean TBR_max_ in AA was slightly, but not significantly (*P* = .18), higher in PLWH (AA = 2.07, IQR 1.97-2.32) than uninfected people (AA = 2.01, IQR 1.85-2.16). No between-group differences were found in other arterial segments, as well (Figure 1 and Supplemental Table 3). However, on multivariable analyses (Table 2), PLWH had a higher risk of increased FDG uptake in the AA (Model 1: coef. = 0.12; 95%CI 0.01-0.22, *P* = .032, Model 2 coef. = 0.12; 95%CI 0.005-0.24, *P* = .041). The association between HIV status and higher FDG uptake in AA was also confirmed without log-transforming the mean TBR_max_ (Model 1 coef. = 0.25; 95%CI 0.02-0.49, *P* = .034, Model 2 = 0.26; 95%CI 0.01-0.52, *P* = .044).

**Table 2:** Association between HIV status and 18F-FDG measurements

Variable	Whole segment method		Most diseased segment method		Active segments method	
	HIV Coef (95%CI)	*P* value	HIV Coef (95%CI)	*P* value	HIV Coef (95%CI)	*P* value
Model 1^a^						
Log mean TBR_max_ in AA	**0.12** **(0.01; 0.22)**	**.032**	**0.13** **(0.02; 0.23)**	**.022**	**0.10 (0.003; 0.20)**	**.043**
Log mean TBR_max_ in DA	0.06 (− 0.03; 0.15)	.17	0.08 (− 0.04; 0.20)	.17	0.04 (− 0.03; 0.11)	.25
Log mean TBR_max_ in CAs	0.05 (− 0.07; 0.17)	.39	0.11 (− 0.05; 0.27)	.18	0.06 (− 0.02; 0.14)	.17
Model 2^b^						
Log mean TBR_max_ in AA	**0.12** **(0.01; 0.24)**	**.041**	**0.13** **(0.01; 0.25)**	**.032**	**0.11 (0.002; 0.22)**	**.046**
Log mean TBR_max_ in DA	0.07 (− 0.02; 0.17)	.12	0.14 (− 0.01; 0.27)	.033	0.05 (− 0.03; 0.13)	.18
Log mean TBR_max_ in CAs	0.04 (− 0.10; 0.17)	.58	0.07 (− 0.11; 0.25)	.42	0.04 (− 0.05; 0.13)	.40

Multivariable linear regression . Whole segment method: averaged TBR_max_ for each slice of the vessel of interest. Most diseased segment method: TBR_max_ of the arterial slice with the highest 18F-FDG uptake in the vessel of interest, averaged with the slice above and below. Active segment method: averaged TBR_max_ of active segments with TBRmax ≥ 1.6, in the vessel of interest.

*AA*, Ascending aorta; *ACC/ASCVD*, American College of Cardiology/Atherosclerotic cardiovascular disease; *18 F-FDG*, 18F-Fluorodeoxyglucose; *LDL*, Low Density Lipoprotein; *TBR*, Target background ratio

^a^Adjusted for body mass index, creatinine, ACC/ASCVD prediction tool, statin use

^b^Adjusted for gender, body mass index, LDL-cholesterol and creatinine

Among the other variables, only the body mass index was associated with FDG uptake in the aorta (Supplemental table 4).

### Biomarkers and HIV Status

Supplemental Table 5 shows the levels of plasma biomarkers according to HIV status. PLWH were more likely to have higher levels of interleukin-10 (IL-10), tumor necrosis factor-α (TNFα), Interferon-γ (IFN-γ), intercellular adhesion molecule-1 (ICAM-1), and vascular cell adhesion molecule-1 (VCAM-1) than uninfected individuals. Conversely, PLWH were also more likely to have lower levels of C-reactive protein than uninfected individuals. On multivariable analysis, HIV infection was associated with a higher value of IL-10, INFγ, and VCAM-1 than uninfected individuals (Table 3).

**Table 3:** Association between HIV status and biomarker levels

Outcome variable	Model 1^a^		Model 2^b^	
	HIV Coef (95%CI)	*P* value	HIV Coef (95%CI)	*P* value
General markers of inflammation				
Log D-dimer	0.38 (− 0.36; 1.42)	.46	0.59 (− 0.49; 1.68)	.27
Fibrinogen	− 1.22 (− 5.61; 3.17)	.57	− 1.25 (− 5.80; 3.31)	.58
Log CRP	− 0.61 (− 1.27; 0.05)	.07	− 0.63 (− 1.34; 0.08)	.08
Inflammatory cytokines				
Log IL-6	0.06 (− 0.36; 0.52)	.72	0.13 (− 0.34; 0.62)	.57
Log IL-10	0.83 (0.34; 1.32)	.001	0.87 (0.38; 1.37)	.001
Log IL-18	0.10 (− 0.24; 0.45)	.54	0.05 (− 0.32; 0.43)	.78
Log TNFα	0.47 (− 0.05; 0.99)	.07	0.40 (− 0.18; 0.98)	.17
Log INFγ	0.90 (0.32; 1.47)	.003	0.70 (− 0.01; 1.41)	.05
Intracellular adhesion molecules				
Log ICAM-1	0.20 (− 0.04; 0.44)	.09	0.16 (− 0.10; 0.42)	.21
Log VCAM-1	0.75 (0.42; 1.08)	< .001	0.82 (0.48; 1.17)	< .001
Markers of macrophage activation				
Log sCD163	0.28 (− 0.82; 0.88)	.36	0.28 (− 0.35; 0.92)	.38
Log sCD14	0.04 (− 0.44; 0.53)	.86	0.02 (− 0.43; 0.48)	.91

Multivariable linear regression

^a^Adjusted for body mass index, creatinine, ACC/ASCVD prediction tool, statin use

^b^Adjusted for gender, body mass index, LDL and creatinine

*ACC/ASCVD*, American College of Cardiology/Atherosclerotic cardiovascular disease; *CRP*, C-reactive protein; *IL*, interleukin; *TNF*, tumor necrosis factor; *ICAM-1*, intercellular adhesion molecule-1; *IFN*, interferon; *LDL*, low-density lipoprotein; *sCD*, soluble cluster of differentiation; *VCAM-1* vascular cell adhesion molecule-1

### Biomarkers and Arterial FDG Uptake

On univariable linear regression restricted to PLWH, we did not find any association between biomarkers and ^18^F-FDG uptake in the 3 study vessels (Supplemental Table 6). In uninfected people (Supplemental Table 7), we found a statistically significant inverse linear association between (1) IL-18 and mean TBR_max_ in AA (Coef = − 0.01; 95%CI − 0.28; -0.01, *P* = .036) and (2) between soluble cluster of differentiation (sCD14) and mean TBR_max_ in CA (Coef = − 0.09; 95%CI − 0.17; − 0.005, *P* = .039). However, these associations were not confirmed on multivariable analyses, with level of sCD14 showing only a trend towards to an association with TBR_max_ in CA (Model 1: Coef = − 0.10; 95%CI − 0.20; − 0.0001, *P* = .050; Model 2: Coef = − 0.09; 95%CI − 0.20; − 0.010, *P* = .073).

### Clinical Events

**Figure 2:**
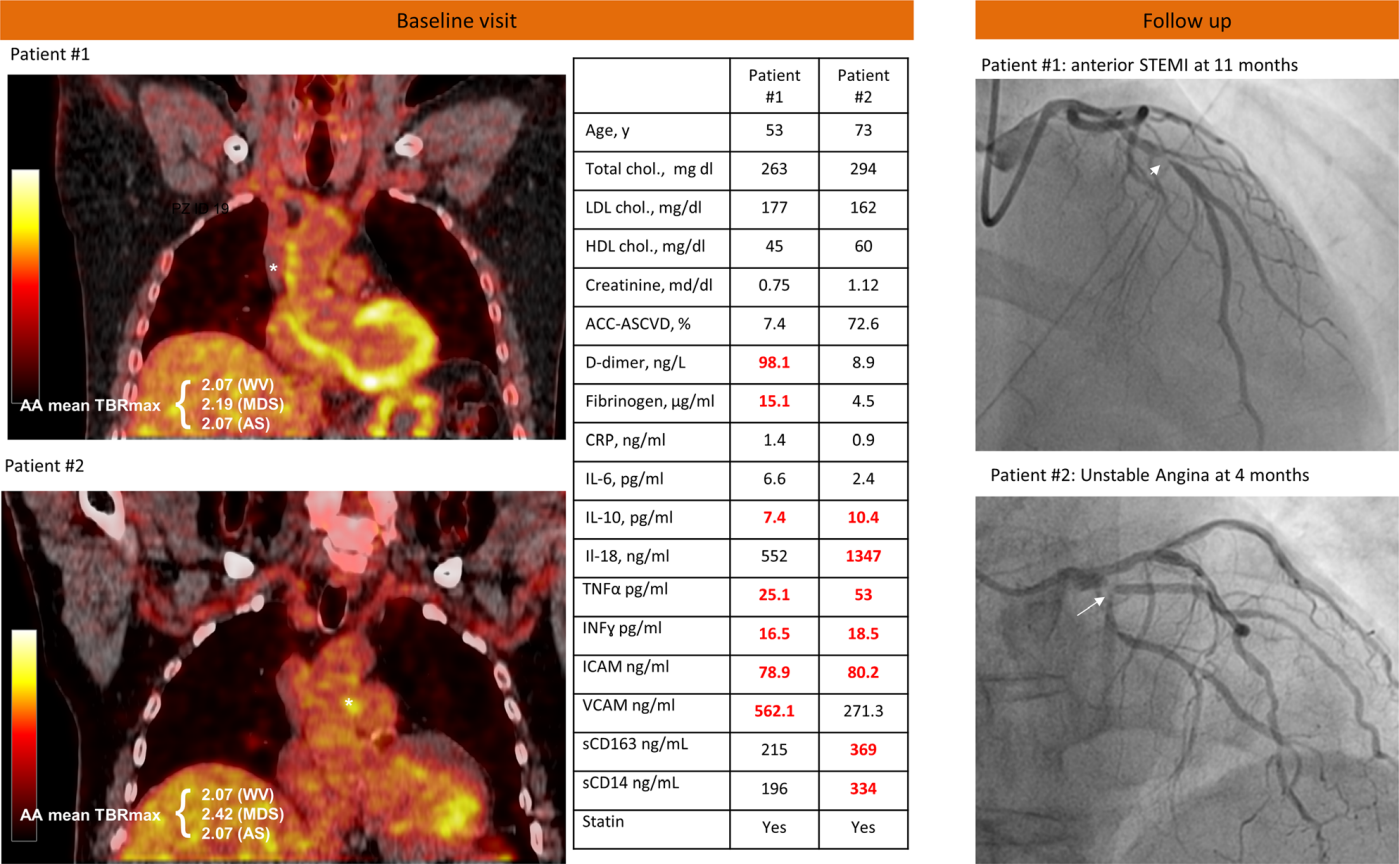
Baseline FDG-PET scans and biomarker levels in 2 HIV patients with clinical events during follow-up. Left panel shows FDG-PET/CT scans from 2 HIV patients that experienced acute coronary events during follow-up. Mean TBR measurements in the ascending aorta were similar (asterisks indicate the area of highest FDG uptake) despite a very different risk profile as assessed by ACC/ASCVD function (table). Red values in the table indicate biomarker levels above the median value of the control group. Right panels show culprit lesion in left anterior descendent artery (arrowhead) and proximal circumflex artery (arrow) for patients 1 and 2, respectively. *ACC/ASCVD*, American College of Cardiology/Atherosclerotic cardiovascular disease; *AS*, active segments methods; *CRP*, C-reactive protein; *IL*, interleukin; *ICAM-1*, intercellular adhesion molecule-1; *IFN*, interferon; *MDS*, most diseased segment; *sCD*, soluble cluster of differentiation; *TBR*, target-to-background ratio; *TNF*, tumor necrosis factor; *VCAM-1*, vascular cell adhesion molecule-1; *WVM*, whole vessel methods

Although not powered to formally assess for differences in clinical outcome between the two groups, during a 1-year follow-up period, 2 patients in the HIV group experienced cardiovascular events. The first patient had an anterior STEMI, due to left anterior descending artery obstruction treated with primary angioplasty. The second patient was admitted for new onset and progressive unstable angina. Coronary angiogram showed a severe obstruction of the proximal left circumflex artery that was treated by percutaneous coronary intervention. Figure 2 shows that at baseline visit their 10-y estimated risk was remarkably different (7.4 vs. 72.6%) while they showed a similar increased FDG uptake in the AA wall. Compared to the median values of the study control group, they both had increased values of IFN-γ and IL-10. Only the first patient showed an increased value of VCAM-1. Although they were both on statin therapy, their cholesterol levels were not on target.

## Discussion

The main findings of this prospective study of 40 individuals with high cardiovascular risk and no known cardiovascular disease are as follows: (1) HIV infection was identified as an independent predictor of increased AA wall inflammation as assessed by ^18^ FDG-PET, and (2) HIV infection was also found to be an independent predictor of increased levels of inflammatory cytokines such as IL-10 and INF-γ, as well as of markers of activated endothelium such as VCAM-1.

Several studies have shown that PLWH have a 1.5- to 2-fold increased risk of MI^4^ and stroke,^5^ compared to uninfected people. The link between HIV infection and cardiovascular disease is multifactorial and relies on the interplay between many factors, including chronic inflammation and immune activation, despite effective ART.

Previous studies that have investigated patterns of arterial ^18^F-FDG-PET in PLWH and control subjects have shown conflicting results.^16^,^17^,^24^ Subramanian et al.^16^ showed, in a cross-sectional study, that FDG uptake in the AA was higher in 27 participants with HIV compared with 27 subjects with no known atherosclerotic disease matched for age, sex, and Framingham risk score (mean FRS = 6.5). In that study, subjects in the control group were not prospectively enrolled. In another retrospective cross-sectional study, Lawal et al.^24^ enrolled 121 PLWH and 121 controls matched for age and gender. The study population was relatively young (range 18-40 years) and had neither known cardiovascular disease nor traditional cardiovascular disease risk factors. The authors found a slightly higher mean TBRmax in AA among PLWH than uninfected people (mean 1.22 ± 0.20 vs. 1.12 ± 0.14, *P* < .001).

Both studies, however, share the same limitations due to their retrospective design. Moreover, FDG-PET acquisition protocols were not optimized for vascular imaging. In the study by Subramanian et al.,^16^ patients with HIV infection were scanned according to recommended uptake time^19^ (≥ 90 min from tracer administration to image acquisition), while in the retrospectively enrolled control group, the uptake time was significantly lower as per clinical evaluation (usually 60 min). This is important since arterial TBR has been shown to increase over time due to a faster washout of FDG in the lumen (blood signal) than in the arterial wall. Therefore, between-group differences in terms of acquisition time could have favored higher values of TBR in PLWH. Yet, the short uptake time (60min) applied in the study by Lawal et al.^24^ might have hampered TBR measurements in arterial wall due to a still high blood signal that may spill over in a thin aorta wall such that of young people (mean age 34.9 ± 5.5).

Unlike the previous studies, Kudnsen et al.^17^ prospectively enrolled 26 patients with HIV and 25 healthy volunteers with no known cardiovascular disease or diabetes. Patients underwent the same FDG-PET protocol, including the prolonged uptake time (3 hours) recommended for vascular imaging. Although HIV patients disclosed a higher FRS-coronary artery disease score (FRS-CHD) than controls, the risk profile of the study population was overall low (FRS-CHD = 7.8 vs. 4.1; *P* = .03). This observation could partly explain why there was no between-group differences in terms of TBR in any of the arterial region targeted (AA, DA, abdominal aorta, carotid arteries) in that study.

In the present prospective study, we found that PLWH had numerically higher mean TBRmax in the AA than controls. However, the between-group difference in arterial inflammation was lower than expected. Compared to the study from Subramanian et al.,^16^ this lower between-group difference relies on both a lower TBR_max_ mean value in HIV group (2.23 vs. 2.19) and a higher TBR_max_ mean value in the control group (1.89 vs. 2.03). The former finding may be explained by the higher percentage of statin therapy in our study (50% vs.0), and the latter may be due to both a higher cardiovascular risk profile and a longer acquisition time in the control group of our study. Nonetheless, after adjustment for potential confounding factors, we observed that HIV status was independently associated with higher ^18^FDG uptake in the AA wall. This result was consistent across all recommended methods for TBR measurement. The difference between univariable and multivariable analyses was likely related to the between-groups differences in terms of body mass index since this latter variable, in keeping with previous investigation,^25^ was independently associated with FDG uptake in the aorta.

Therefore, the results from our study are consistent with previous work and provide further evidence that in patients with high cardiovascular risk HIV status should be considered as an additional cardiovascular risk factor because of its association with arterial inflammation. This is important because many patients with HIV have traditional risk factors for cardiovascular disease^3^; however, this association is clinically under-recognized.^10^

Accordingly, care delivery systems should focus on aggressive preventative strategies to correct modifiable risk factors in PLWH.

In the present study we also sought to evaluate whether PLWH, compared to uninfected people, show different patterns of biomarkers involved in the pathways of atherosclerosis.

We found, on multivariable analysis, that patients with HIV had higher levels of IL-10, INF-γ, and VCAM-1. This pattern is consistent with HIV status. Indeed, in viral infections, INF-γ represents a key pro-inflammatory cytokine secreted by lymphocyte T helper-1 cells. INF-γ subsequently favors activation of macrophages, natural killer T cells, dendritic cells, and a second wave of T-cell activation. IFN-γ can also activate vascular smooth muscle cells and promote the recruitment of immune cells by inducing the expression of adhesion molecules, such as VCAM-1, in endothelial cells.^26^ IL-10 is one of the most important anti-inflammatory cytokines.^27^ As an anti-inflammatory cytokine, its high levels could represent a counteraction to chronic inflammatory immune activation associated with HIV infection.

Although it is known that inflammatory and immune responses are also involved in the development and progression of atherosclerosis,^28^ in our study, we did not find any association between biomarkers and the degree of arterial inflammation.

Among PLWH, unlike previous studies,^16^ we did not find any significant associations between sCD163 (a marker of macrophage activation) and ^18^FDG uptake in the AA wall, despite the known associations of arterial FDG uptake and the concentration and metabolic activity of macrophages in atherosclerotic plaques.^14^ The reason for this finding is unknown. The present study could be underpowered to detect this kind of association. Factors, other than the extent of atherosclerosis, such as the duration and type of ART^29^ could also influence the plasma levels of macrophage activation markers. Finally, other glucose metabolizing inflammatory or arterial cells in this high-risk population could contribute to the aorta ^18^F-FDG uptake. The use of other PET tracers in future studies (e.g., ^68^Ga-DOTA- (Tyr3)-octreotate for M1 macrophages^30^ and ^18^F-Sodium fluoride for microcalcification^31^) could also potentially help to more precisely compare mechanisms of atherosclerosis that are most active in PLWH.

### Limitations

The results of the present study should be interpreted with caution in light of some limitations, including a relatively small sample size. Indeed, the present study was powered only to detect differences in term of mean TBR_max_ in AA and we have could have missed other significant relationships. FDG uptakes in DA and CA were also higher in HIV patients than controls but these findings were not statistically significant even controlling for unbalanced confounders. We chose TBR_max_ in AA as primary endpoint since it is the most studied artery district in HIV patients and FDG uptake in AA has been associated with an increased risk of future events.^15^ Yet, to our knowledge, this is the first study to report the relationship between 18F–FDG uptake in the thoracic aorta and HIV infection in patients with moderate-to-high cardiovascular risk, compared to a prospectively enrolled control group with traditional cardiovascular risk factors.

## Conclusions

In this prospective, cross-sectional study of patients with a moderate–high cardiovascular risk profile, HIV status was identified as an independent predictor of increased AA wall inflammation. PLWH also had an independent risk of increased level of IFN-γ, IL-10, and VCAM-1.

## New Knowledge Gained

In patients with moderate-to-high cardiovascular risk, HIV status was an independent predictor of increased ascending aortic wall inflammation as assessed by ^18^F-Fluorodeoxyglucose-positron emission tomography imaging.

## Electronic supplementary material

Below is the link to the electronic supplementary material.

Electronic supplementary material 1 (DOCX 142 kb)

Electronic supplementary material 2 (PPTX 5090 kb)

Electronic supplementary material 3 (MP3 4441 kb)

## Abbreviations

AA: Ascending aorta
ACC/ASCVD: American College of Cardiology/atherosclerotic cardiovascular disease
ART: Antiretroviral therapy
CA: Carotid arteries
DA: Descending aorta
^18^F-FDG-PET/CT: Fluorodeoxyglucose-positron emission tomography/computed tomography
MI: Myocardial infarction
PLWH: People living with HIV
SUV: Standardized uptake values
TBR: Target-to-background ratio
