# Supplementary material for: Pattern of arterial inflammation and inflammatory markers in people living with HIV compared with uninfected people
Source: J Nucl Cardiol. 2021 Feb 10;29(4):1566–75. doi: 10.1007/s12350-020-02522-5 (PMC9345795; doi:10.1007/s12350-020-02522-5)
Supplement: Supplementary file 1 — Electronic supplementary material 1 (DOCX 142 kb) [file 12350_2020_2522_MOESM1_ESM.docx]

**Supplemental materi****al**

[**Supplemental Table 1** Biomarkers assays specifications 2](#_Toc56694932)

[**Supplemental Table 2** HIV related laboratory findings and medications 3](#_Toc56694933)

[**Supplemental Table 3** ^18^F-FDG uptake measured within the wall of ascending, descending thoracic aorta and both carotid arteries 4](#_Toc56694934)

[**Supplemental Table 4.** Association between BMI (modelled as continuous variable) and ^18^F-FDG measurements. Multivariable linear regression 5](#_Toc56694935)

[**Supplemental Table 5**. Serum level of study biomarkers according to the HIV status 6](#_Toc56694936)

[**Supplemental Table 6.** Univariable linear regression between biomarkers (independent variable) and arterial FDG uptake (outcome variable) in PLWH 7](#_Toc56694937)

[**Supplemental Table 7.** Univariable linear regression between biomarkers (independent variable) and arterial FDG uptake (outcome variable) in people with no HIV infection 9](#_Toc56694938)

[**Supplemental Figures Legend** 10](#_Toc56694939)

[**Supplemental Figure 1** 11](#_Toc56694940)

# **Supplemental Table 1** Biomarkers assays specifications

| **Assay** | **Marker** | **Manufacturer** | **Analytical Range** | **Coefficient**  **of variation** |
| --- | --- | --- | --- | --- |
| ELISA | Human IL-6 | R&D System | 3.1-300 pg/mL | 4.5% |
| ELISA | Human IL-10 | R&D System | 7.8-500 pg/mL | 6.9% |
| ELISA | Human IL-18 | R&D System | 15.6-1000 pg/mL | 8.3% |
| ELISA | Human C-Reactive Protein | R&D System | 0.78-50 ng/mL | 6.5% |
| ELISA | Human ICAM-1 | R&D System | 1.56-50 ng/mL | 5.5% |
| ELISA | Human IFN-γ | R&D System | 15.6-1000 pg/mL | 5.9% |
| ELISA | Human TFN-α | R&D System | 15.6-1000 pg/mL | 7.6% |
| ELISA | Human VCAM-1 | R&D System | 6.25-200 ng/mL | 7.0% |
| ELISA | Human D-Dimer | CUSABIO | 15.6-1000 ng/mL | <10% |
| ELISA | Human Fibrinogen | Abnova | 2.5-40 µg/mL | 9.9% |
| ELISA | sCD163 | R&D System | 1.56-100 ng/ml | 5.1% |
| ELISA | sCD14 | R&D System | 250-1600 pg/mL | 6.3% |

# **Supplemental Table 2** HIV related laboratory findings and medications

| **Variable** | **PLWH**  **n=20** |
| --- | --- |
| HIV-RNA copies/ml <20 | 18 (90) |
| HIV-DNA copies/ml, median (25^th^-75^th^) | 44 (25-93) |
| CD4 count, /mm^2^ | 871 (580-1060) |
| CD4/CD8 ratio, median (25^th^-75^th^) | 0.73 (0.5-1.11) |
| ART duration, y median (25^th^-75^th^) | 20.2 (11.2-24.4) |
| Current PI (%) | 6 (30) |
| Current NRTI (%) | 19 (95) |
| Current PIs+NRTI (%) | 5 (25) |

PI= protease inhibitors; NRTI: non–nucleoside reverse transcriptase inhibitor

# **Supplemental Table 3** ^18^F-FDG uptake measured within the wall of ascending, descending thoracic aorta and both carotid arteries

| **Variable** | **HIV**  **+** | **HIV**  **-** | **P value** |
| --- | --- | --- | --- |
| **No. of patients** | **n = 20** | **n = 20** |  |
| *Whole segment method* |  |  |  |
| Mean TBR_max_ in AA | 2.07 (1.97-2.32) | 2.01 (1.85-2.16) | 0.18 |
| Mean TBR_max_ in DA | 1.98 (1.79-2.07) | 1.88 (1.77-2.07) | 0.48 |
| Mean TBR_max_ in CAs | 1.60 (1.47-1.93) | 1.62 (1.42-1.70) | 0.52 |
| *Most segment diseased method* |  |  |  |
| Mean TBR_max_ in AA | 2.23 (2.03-2.48) | 2.07 (1.92-2.29) | 0.12 |
| Mean TBR_max_ in DA | 2.39 (2.06-2.60) | 2.29 (1.99-2.52) | 0.33 |
| Mean TBR_max_ in CAs | 1.94 (1.71-2.41) | 1.87 (1.65-2.11) | 0.42 |
| *Active segments methods* |  |  |  |
| Mean TBR_max_ in AA | 2.08 (1.97.2.32) | 2.01 (1.85-2.16) | 0.20 |
| Mean TBR_max_ in DA | 2.02 (1.85-2.11) | 1.91 (1.81-2.11) | 0.57 |
| Mean TBR_max_ in CAs | 1.83 (1.75-2.09) | 1.80 (1.73-1.87) | 0.33 |

AA: ascending aorta; CAs: carotid arteries; DA. Descending Aorta, TBR: target background ratio

# **Supplemental Table 4.** Association between BMI (modelled as continuous variable) and ^18^F-FDG measurements. Multivariable linear regression

| **Variable** | **Whole segment method** | | **Most diseased segment method** | | **Active segments method** | |
| --- | --- | --- | --- | --- | --- | --- |
|  | **BMI**  **Coef (95%CI)** | **p**  **value** | **BMI**  **Coef (95%CI)** | **p**  **value** | **BMI**  **Coef (95%CI)** | **p**  **value** |
| Model 1* | | | | | | |
| Log mean TBRmax in AA | **0.02**  **(0.004;0.038)** | **0.015** | **0.03**  **(0.008;0.041)** | **0.004** | **0.02**  **(0.004;0.034)** | **0.017** |
| Log mean TBRmax in DA | **0.02**  **(0.003;0.031)** | **0.022** | **0.02**  **(0.002;0.040)** | **0.029** | **0.01**  **(0.003;0.026)** | **0.016** |
| Log mean TBRmax in CAs | 0.007  (-0.001;0.026) | 0.46 | 0.02  (-0.007;0.044) | 0.15 | 0.004  (-0.008;0.18) | 0.45 |
| Model 2† | | | | | | |
| Log mean TBRmax in AA | **0.02**  **(-0.004;0.035)** | **0.014** | **0.02**  **(0.008;0.040)** | **0.004** | **0.018 (0.004;0.032)** | **0.016** |
| Log mean TBRmax in DA | **0.02**  **(0.006;0.031)** | **0.004** | **0.02**  **(-0.005;0.041)** | **0.011** | **0.02**  **(0.006;0.027)** | **0.003** |
| Log mean TBRmax in CAs | 0.014  (-0.003;0.032) | 0.12 | **0.03**  **(0.003;0.050)** | **0.027** | 0.006  (-0.006;0.019) | 0.31 |

* Adjusted for HIV, creatinine, ACC/ASCVD prediction tool, statin use

† Adjusted for gender, HIV, LDL and creatinine

Whole segment method: averaged TBRmax for each slice of the vessel of interest.

Most diseased segment method: TBRmax of the arterial slice with the highest 18F-FDG uptake in the vessel of interest, averaged with the slice above and below.

Active segment method: averaged TBRmax of active segments with TBRmax ≥1.6, in the vessel of interest.

AA = Ascending aorta, ACC/ASCVD: American College of Cardiology/Atherosclerotic cardiovascular disease, BMI: body mass index, ^18^ F-FDG: ^18^F-Fluorodeoxyglucose, LDL = Low Density Lipoprotein, TBR: Target background ratio

# **Supplemental Table 5**. Serum level of study biomarkers according to the HIV status

| **Variable** | **HIV**  **+** | **HIV**  **-** | **P value** |
| --- | --- | --- | --- |
| **No. of patients** | **n = 20** | **n = 20** |  |
| *General markers of inflammation* |  |  |  |
| D-dimer ng/L | 29.0 (15.8-58.4) | 20.8(5.4-62.1) | 0.35 |
| Fibrinogen µg/ml | 10.4 (8.3-14.6) | 14.4(9-19.2) | 0.14 |
| CRP ng/ml | 1.3 (0.7-5.1) | 4.5(1.6-7.5) | 0.016 |
| *Inflammatory cytochines* |  |  |  |
| IL-6 pg/mL | 3.8 (2.2-6.5) | 4.12(1.99-4.78) | 0.43 |
| IL-10 pg/ml | 14.2 (13.6-22.7) | 5.4(3.3-12.7) | 0.001 |
| IL-18 pg/ml | 511.0 (419-753) | 557.8(369.4-765.4) | 0.9 |
| TNFα pg/ml | 48.0 (27.3-57.8) | 24.13(19.4-46.9) | 0.08 |
| INFɣ pg/ml | 13.7 (9.9-17.0) | 7.8(5.2-11.2) | 0.03 |
| *Intracellular adhesione molecules* |  |  |  |
| ICAM ng/ml | 103.0 (82.5-132.7) | 78.5(70.6-98.2) | 0.02 |
| VCAM ng/ml | 652.7 (546.9-714.1) | 286.3(234.3-369.4) | <0.001 |
| *Markers of macrophage activation* |  |  |  |
| sCD163 ng/ml | 345.3 (211.6-567.9) | 338.9(222.6-446.7) | 0.7 |
| sCD14 ng/ml | 248.1 (225.7-260.7) | 243.9(201.3-270.3) | 0.9 |

CRP: C-reactive protein, IL =interleukin, TNF: tumor necrosis factor, ICAM-1: Intercellular adhesion molecule-1: IFN: Interferon, sCD: soluble cluster of differentiation; VCAM-1: vascular cell adhesion molecule-1

# **Supplemental Table 6.** Univariable linear regression between biomarkers (independent variable) and arterial FDG uptake (outcome variable) in PLWH

| **Variable** | **Ascending Aorta** | | **Descending Aorta** | | **Carotid arteries** | |
| --- | --- | --- | --- | --- | --- | --- |
|  | **Log mean TBR_max_**  (whole vessel method) | | | | | |
|  | **Coeff (95%CI)** | **p**  **value** | **Coeff.**  **(95%CI)** | **p**  **value** | **Coeff (95%CI))** | **p**  **value** |
| *HIV retaliated findings* |  |  |  |  |  |  |
| Log HIV-DNA copies/ml | 0.01 (-0.057;0.08) | 0.73 | 0.001 (-0.046; 0.05) | 0.96 | 0.02 (-0.07;0.11) | 0.66 |
| CD4 count, cells/ml | 0.0001 (-0.0003-0.0002) | 0.45 | 0.00003(-0.0002;0.0001) | 0.74 | 0.0003 (-0.0003;0.0003) | 0.81 |
| CD4/CD8 ratio | 0.01 (-0.20;0.23) | 0.88 | 0.034 (-0.11;0.18) | 0.63 | 0.034 (-0.30;0.23) | 0.79 |
| *General markers of inflammation* |  |  |  |  |  |  |
| Log D-dimer ng/L | 0.04 (-0.039;0.13) | 0.29 | 0.04 (-0.02;0.09) | 0.18 | 0.03 ( -0.07-0.14) | 0.53 |
| Fibrinogen mg/dl | -0.01 (-0.02:0.01) | 0.72 | 0.01 (-0.01;0.01) | 0.79 | -0.01 (-0.02;0.01) | 0.46 |
| Log CRP mg/dl | -0.02 (-0.10;0.06) | 0.61 | -0.008 (-0.06;0.05) | 0.76 | 0.04 (-0.06-0.14) | 0.38 |
| *Inflammatory cytochines* |  |  |  |  |  |  |
| Log IL-6 pg/ml | 0.09 (-0.01;0.20) | 0.09 | 0.06 (-0.02;0.13) | 0.1 | -0.04 (-0.19;0.10) | 0.56 |
| Log IL-10 pg/mL | 0.11 -0.05;-0.27) | 0.16 | 0.04 (-0.07;0.16) | 0.42 | -0.02 (-0.23;0.19) | 0.85 |
| Log IL-18 pg/mL | -0.02 (-0.17;0.13) | 0.80 | 0.01 (-0.10;0.11) | 0.90 | -0.02 (-0.21;0.18) | 0.83 |
| Log TNFα pg/mL | -0.04 (-0.14;0.07) | 0.46 | 0.01 (-0.06;0.09) | 0.68 | -0.04 (-0.15;0.07) | 0.48 |
| Log INFɣ pg/mL | 0.04 (-0.07;0.16) | 0.44 | 0.04 (-0.041;0.12) | 0.33 | 0.10 (-0.038;0.24) | 0.15 |
| *Intracellular adhesione molecules* |  |  |  |  |  |  |
| Log ICAM-1 ng/mL | 0.10 (-0.11;0.31) | 0.32 | 0.08 (-0.04;0.21) | 0.19 | -0.06 (-0.32;0.20) | 0.63 |
| Log VCAM-1 ng/mL | 0.12 (-.34; 0.09) | 0.25 | 0.01 (-0.16;0.14) | 0.87 | -0.17 (-0.43;0.09) | 0.19 |
| *Markers of macrophage activation* |  |  |  |  |  |  |
| Log sCD163 ng/mL | -0.04 (-0.12;0.03) | 0.26 | -0.01 (-0.07;0.40) | 0.58 | -0.07 (-0.16;0.02) | 0.15 |
| Log sCD14 ng/mL | 0.03 (-0.09;0.15) | 0.63 | -0.03 (-0.1,0.4) | 0.41 | 0.05 (-0.11-0.21) | 0.53 |

CRP: C-reactive protein, IL =interleukin, TNF: tumor necrosis factor, ICAM-1: Intercellular adhesion molecule-1: IFN: Interferon, sCD: soluble cluster of differentiation; VCAM-1: vascular cell adhesion molecule-1

# **Supplemental Table 7.** Univariable linear regression between biomarkers (independent variable) and arterial FDG uptake (outcome variable) in people with no HIV infection

| **Variable** | **Ascending Aorta** | | **Descending Aorta** | | **Carotid arteries** | |
| --- | --- | --- | --- | --- | --- | --- |
|  | **Log mean TBR_max_**  (whole vessel method) | | | | | |
|  | **Coeff**  **(95%CI)** | **p**  **value** | **Coeff.**  **(95%CI)** | **p**  **value** | **Coeff**  **(95%CI)** | **p**  **value** |
| *General markers of inflammation* |  |  |  |  |  |  |
| Log D-dimer ng/L | 0.02 (-0.03;0.07) | 0.37 | 0.01 (-0.04;0.05) | 0.81 | 0.01 (-0.03;0.05) | 0.73 |
| Fibrinogen mg/dl | -0.001 (-0.01;0.007) | 0.72 | -0.001 (-0.013;0.012) | 0.88 | -0.03 (-0.01;0.08) | 0.56 |
| Log CRP mg/dl | 0.005 (-0.10;0.11) | 0.92 | 0.002 (-0.11:0.11) | 0.97 | -0.03 (-0.12;0.07) | 0.53 |
| *Inflammatory cytochines* |  |  |  |  |  |  |
| Log IL-6pg/mL | -0.05 (-0.19;0.08) | 0.43 | -0.05 (-0.19;0.08) | 0.41 | 0.05 (-0.07;0.17) | 0.39 |
| Log IL-10 pg/mL | 0.02 (-0.08;0.13) | 0.56 | -0.02 (-0.13;0.08) | 0.69 | 0.007 (-0.09;0.10) | 0.87 |
| **Log IL-18 pg/mL** | **-0.01(-0.28;-0.01)** | **0.036** | -0.13 (-0.27;0.06) | 0.059 | -0.06 (-0.19;0.07) | 0.33 |
| Log TNFα pg/mL | -0.02 (-0.12;0.09) | 0.77 | -0.009 (-0.12;0.10) | 0.86 | -0.02 (0.12;0.07) | 0.60 |
| Log INFɣ pg/mL | -0.01 (-0.08;0.06) | 0.79 | 0.01(0.63;0.08) | 0.73 | -0.01 (-0.07;0.05) | 0.77 |
| *Intracellular adhesione molecules* |  |  |  |  |  |  |
| Log ICAM ng/mL | -0.07 (-0.38;0.23) | 0.63 | 0.06 (-0.25;0.38) | 0.66 | 0.14 (-0.12;0.41) | 0.27 |
| Log VCAM ng/mL | -0.02 (-0.18;0.14) | 0.79 | 0.04 (-0.12;0.21) | 0.59 | -0.02 (-0.17;0.12) | 0.76 |
| *Markers of macrophage activation* |  |  |  |  |  |  |
| Log sCD163 ng/mL | -0.15 (-0.33;0.31) | 0.10 | -0.18 (-0.37;0.001) | 0.051 | -0.07 (-0.25;0.09) | 0.35 |
| **Log sCD14 ng/mL** | 0.01 (-0.10;0.11) | 0.90 | -0.03 (-0.13;0.08) | 0.60 | **-0.09(-0.17;-0.005)** | **0.039** |

CRP: C-reactive protein, IL =interleukin, TNF: tumor necrosis factor, ICAM-1: Intercellular adhesion molecule-1: IFN: Interferon, sCD: soluble cluster of differentiation; VCAM-1: vascular cell adhesion molecule-1

# **Supplemental Figures Legend**

**Supplemental Figure** 1 Study flow chart

# **Supplemental Figure 1**
