# Supplementary material for: Pattern of arterial inflammation and inflammatory markers in people living with HIV compared with uninfected people
Source: J Nucl Cardiol. 2021 Feb 10;29(4):1566–75. doi: 10.1007/s12350-020-02522-5 (PMC9345795; doi:10.1007/s12350-020-02522-5)
Supplement: Supplementary file 2 — Electronic supplementary material 2 (PPTX 5090 kb) [file 12350_2020_2522_MOESM2_ESM.pptx]

## Slide 1
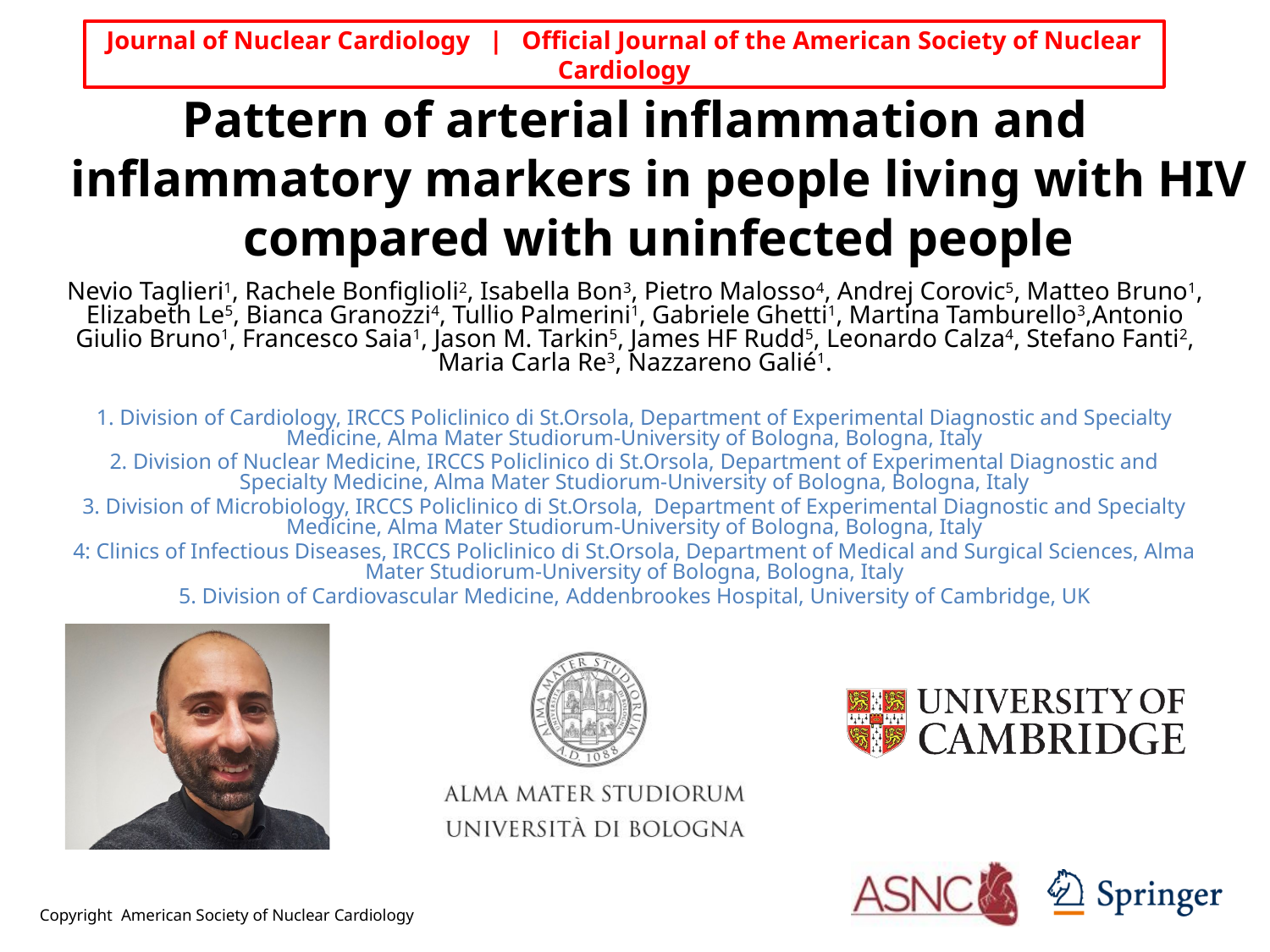

Journal of Nuclear Cardiology | Official Journal of the American Society of Nuclear Cardiology
# Pattern of arterial inflammation and inflammatory markers in people living with HIV compared with uninfected people
Nevio Taglieri1, Rachele Bonfiglioli2, Isabella Bon3, Pietro Malosso4, Andrej Corovic5, Matteo Bruno1, Elizabeth Le5, Bianca Granozzi4, Tullio Palmerini1, Gabriele Ghetti1, Martina Tamburello3,Antonio Giulio Bruno1, Francesco Saia1, Jason M. Tarkin5, James HF Rudd5, Leonardo Calza4, Stefano Fanti2, Maria Carla Re3, Nazzareno Galié1.
1. Division of Cardiology, IRCCS Policlinico di St.Orsola, Department of Experimental Diagnostic and Specialty Medicine, Alma Mater Studiorum-University of Bologna, Bologna, Italy
2. Division of Nuclear Medicine, IRCCS Policlinico di St.Orsola, Department of Experimental Diagnostic and Specialty Medicine, Alma Mater Studiorum-University of Bologna, Bologna, Italy
3. Division of Microbiology, IRCCS Policlinico di St.Orsola, Department of Experimental Diagnostic and Specialty Medicine, Alma Mater Studiorum-University of Bologna, Bologna, Italy
4: Clinics of Infectious Diseases, IRCCS Policlinico di St.Orsola, Department of Medical and Surgical Sciences, Alma Mater Studiorum-University of Bologna, Bologna, Italy
5. Division of Cardiovascular Medicine, Addenbrookes Hospital, University of Cambridge, UK
Head shot of author
required
Copyright American Society of Nuclear Cardiology

## Slide 2
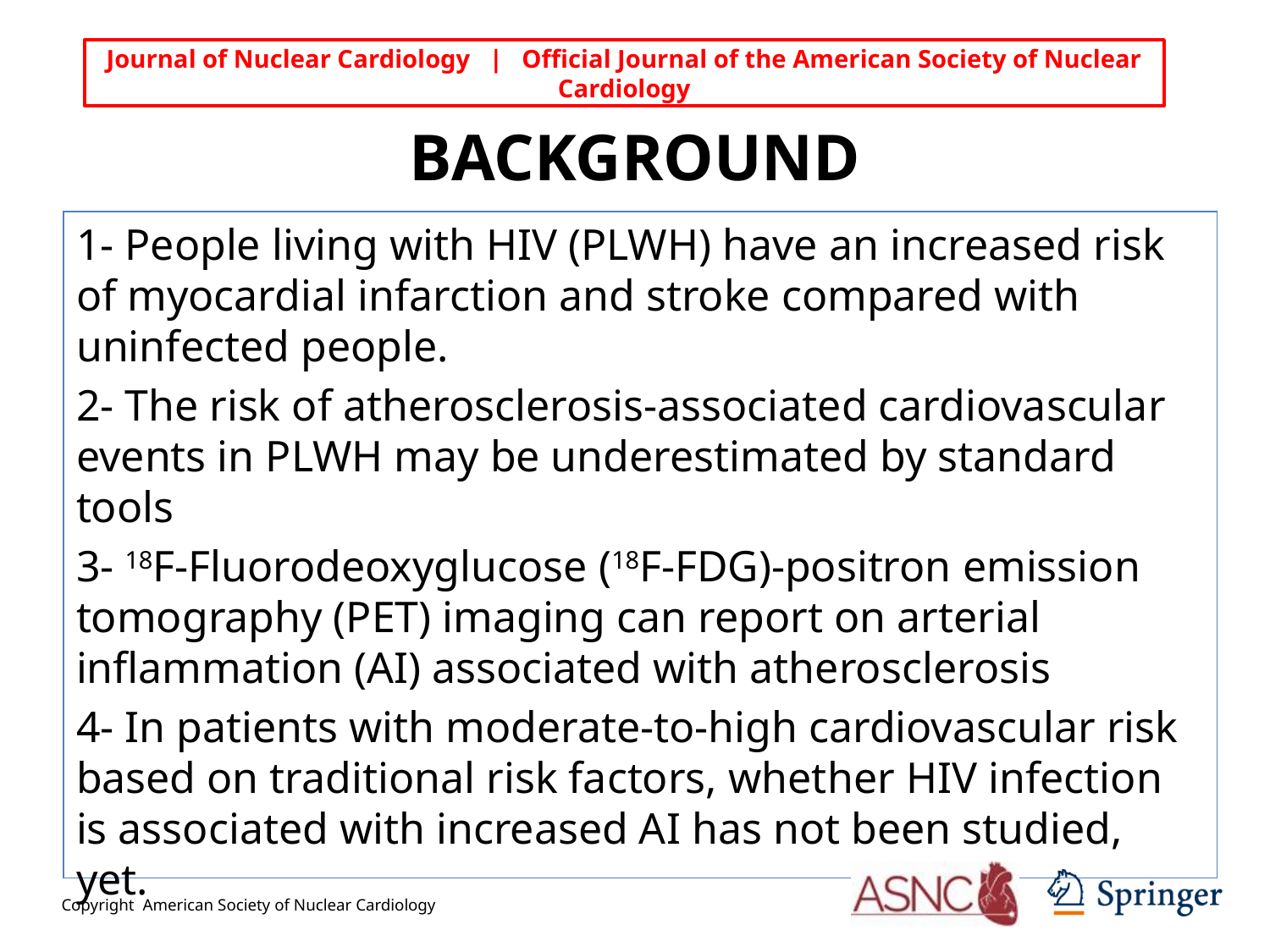

Journal of Nuclear Cardiology | Official Journal of the American Society of Nuclear Cardiology
# BACKGROUND
1- People living with HIV (PLWH) have an increased risk of myocardial infarction and stroke compared with uninfected people.
2- The risk of atherosclerosis-associated cardiovascular events in PLWH may be underestimated by standard tools
3- 18F-Fluorodeoxyglucose (18F-FDG)-positron emission tomography (PET) imaging can report on arterial inflammation (AI) associated with atherosclerosis
4- In patients with moderate-to-high cardiovascular risk based on traditional risk factors, whether HIV infection is associated with increased AI has not been studied, yet.
Copyright American Society of Nuclear Cardiology

## Slide 3
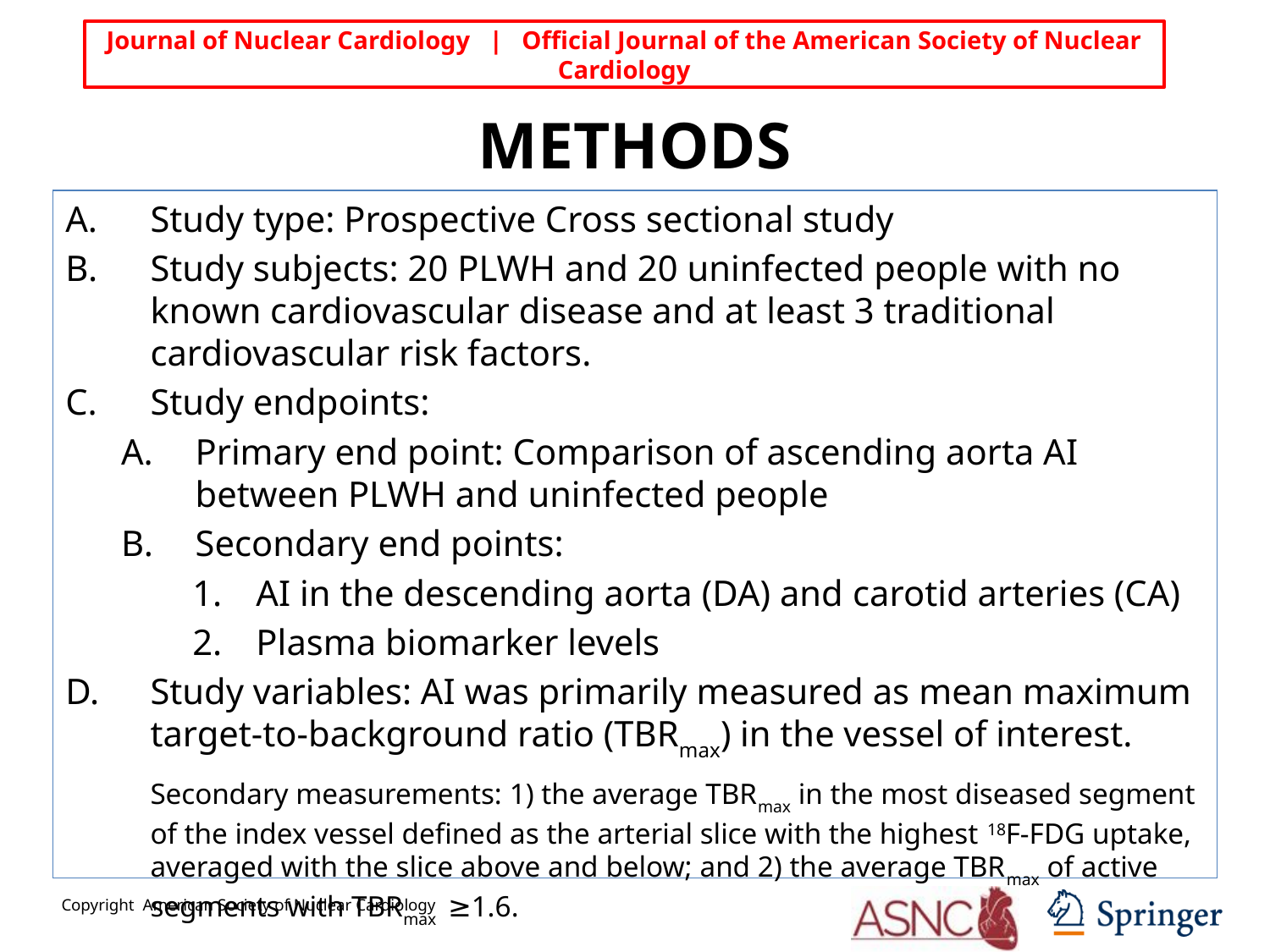

Journal of Nuclear Cardiology | Official Journal of the American Society of Nuclear Cardiology
# METHODS
Study type: Prospective Cross sectional study
Study subjects: 20 PLWH and 20 uninfected people with no known cardiovascular disease and at least 3 traditional cardiovascular risk factors.
Study endpoints:
Primary end point: Comparison of ascending aorta AI between PLWH and uninfected people
Secondary end points:
AI in the descending aorta (DA) and carotid arteries (CA)
Plasma biomarker levels
Study variables: AI was primarily measured as mean maximum target-to-background ratio (TBRmax) in the vessel of interest.
	Secondary measurements: 1) the average TBRmax in the most diseased segment of the index vessel defined as the arterial slice with the highest 18F-FDG uptake, averaged with the slice above and below; and 2) the average TBRmax of active segments with TBRmax ≥1.6.
Copyright American Society of Nuclear Cardiology

## Slide 4
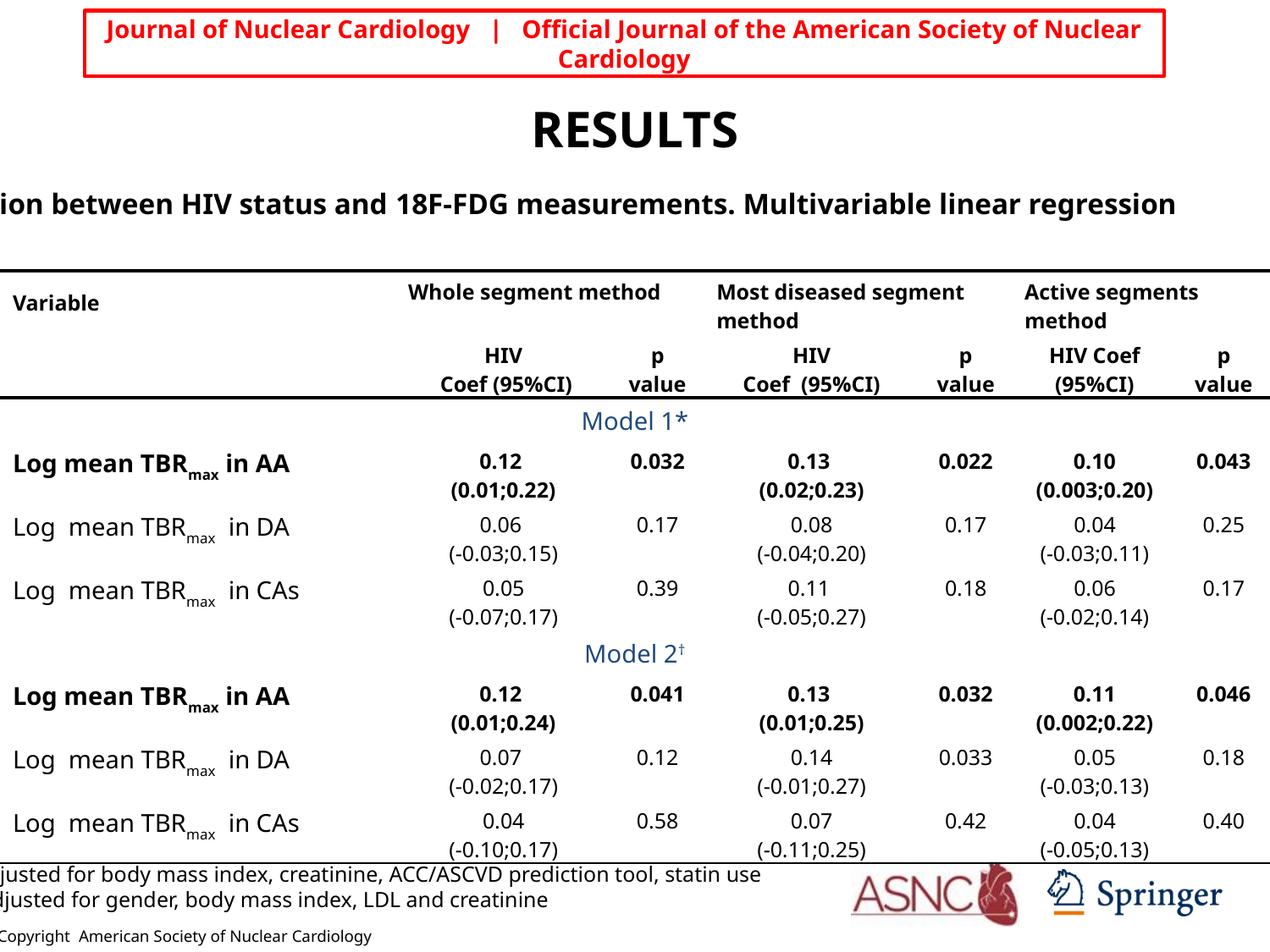

Journal of Nuclear Cardiology | Official Journal of the American Society of Nuclear Cardiology
# RESULTS
Association between HIV status and 18F-FDG measurements. Multivariable linear regression
| Variable | Whole segment method | | Most diseased segment method | | Active segments method | |
| --- | --- | --- | --- | --- | --- | --- |
| | HIV Coef (95%CI) | p value | HIV Coef (95%CI) | p value | HIV Coef (95%CI) | p value |
| Model 1\* | | | | | | |
| Log mean TBRmax in AA | 0.12 (0.01;0.22) | 0.032 | 0.13 (0.02;0.23) | 0.022 | 0.10 (0.003;0.20) | 0.043 |
| Log mean TBRmax in DA | 0.06 (-0.03;0.15) | 0.17 | 0.08 (-0.04;0.20) | 0.17 | 0.04 (-0.03;0.11) | 0.25 |
| Log mean TBRmax in CAs | 0.05 (-0.07;0.17) | 0.39 | 0.11 (-0.05;0.27) | 0.18 | 0.06 (-0.02;0.14) | 0.17 |
| Model 2† | | | | | | |
| Log mean TBRmax in AA | 0.12 (0.01;0.24) | 0.041 | 0.13 (0.01;0.25) | 0.032 | 0.11 (0.002;0.22) | 0.046 |
| Log mean TBRmax in DA | 0.07 (-0.02;0.17) | 0.12 | 0.14 (-0.01;0.27) | 0.033 | 0.05 (-0.03;0.13) | 0.18 |
| Log mean TBRmax in CAs | 0.04 (-0.10;0.17) | 0.58 | 0.07 (-0.11;0.25) | 0.42 | 0.04 (-0.05;0.13) | 0.40 |
* Adjusted for body mass index, creatinine, ACC/ASCVD prediction tool, statin use
† Adjusted for gender, body mass index, LDL and creatinine
Copyright American Society of Nuclear Cardiology

## Slide 5
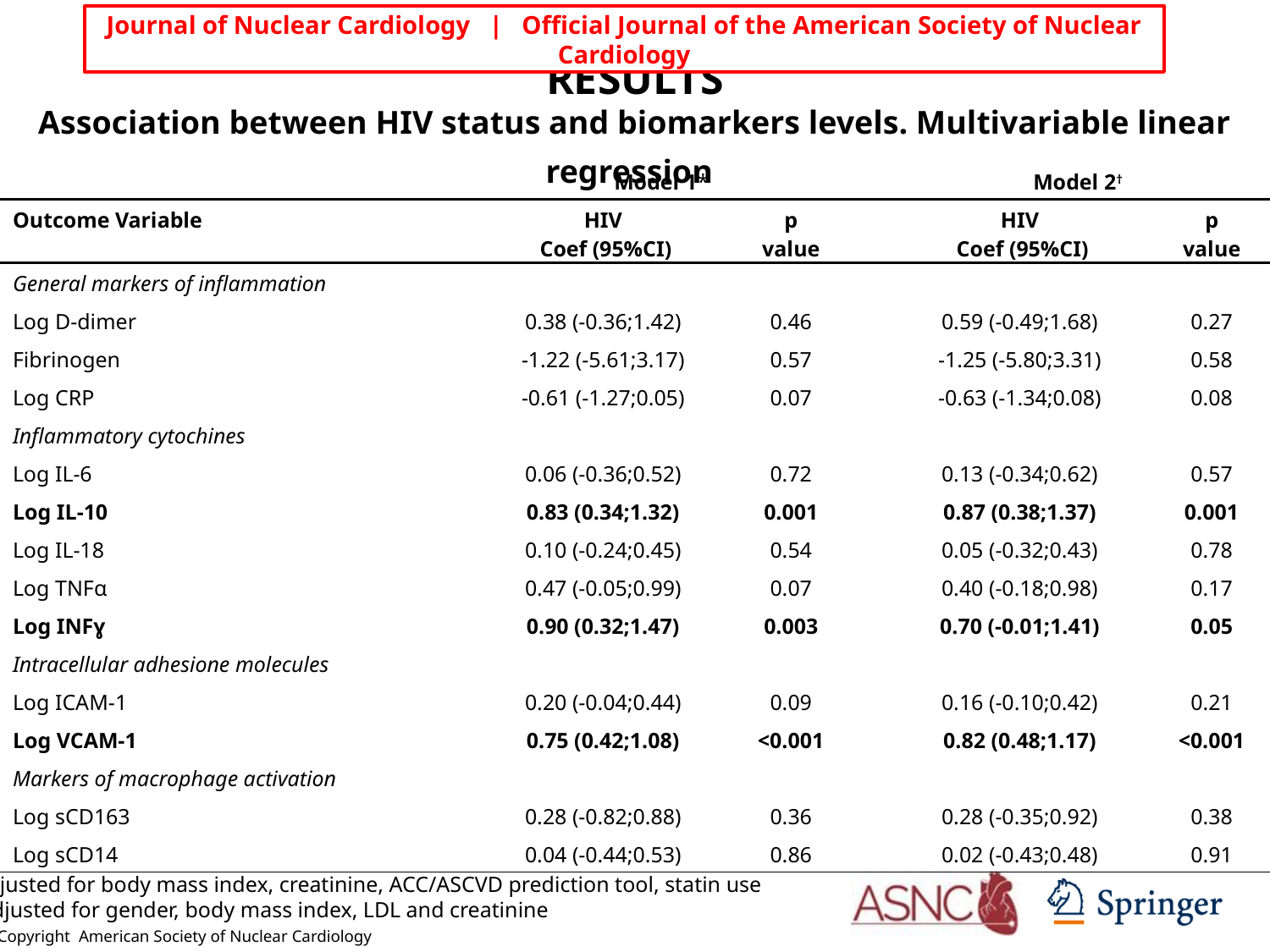

Journal of Nuclear Cardiology | Official Journal of the American Society of Nuclear Cardiology
# RESULTS Association between HIV status and biomarkers levels. Multivariable linear regression
| | Model 1\* | | | Model 2† | |
| --- | --- | --- | --- | --- | --- |
| Outcome Variable | HIV Coef (95%CI) | p value | | HIV Coef (95%CI) | p value |
| General markers of inflammation | | | | | |
| Log D-dimer | 0.38 (-0.36;1.42) | 0.46 | | 0.59 (-0.49;1.68) | 0.27 |
| Fibrinogen | -1.22 (-5.61;3.17) | 0.57 | | -1.25 (-5.80;3.31) | 0.58 |
| Log CRP | -0.61 (-1.27;0.05) | 0.07 | | -0.63 (-1.34;0.08) | 0.08 |
| Inflammatory cytochines | | | | | |
| Log IL-6 | 0.06 (-0.36;0.52) | 0.72 | | 0.13 (-0.34;0.62) | 0.57 |
| Log IL-10 | 0.83 (0.34;1.32) | 0.001 | | 0.87 (0.38;1.37) | 0.001 |
| Log IL-18 | 0.10 (-0.24;0.45) | 0.54 | | 0.05 (-0.32;0.43) | 0.78 |
| Log TNFα | 0.47 (-0.05;0.99) | 0.07 | | 0.40 (-0.18;0.98) | 0.17 |
| Log INFɣ | 0.90 (0.32;1.47) | 0.003 | | 0.70 (-0.01;1.41) | 0.05 |
| Intracellular adhesione molecules | | | | | |
| Log ICAM-1 | 0.20 (-0.04;0.44) | 0.09 | | 0.16 (-0.10;0.42) | 0.21 |
| Log VCAM-1 | 0.75 (0.42;1.08) | <0.001 | | 0.82 (0.48;1.17) | <0.001 |
| Markers of macrophage activation | | | | | |
| Log sCD163 | 0.28 (-0.82;0.88) | 0.36 | | 0.28 (-0.35;0.92) | 0.38 |
| Log sCD14 | 0.04 (-0.44;0.53) | 0.86 | | 0.02 (-0.43;0.48) | 0.91 |
* Adjusted for body mass index, creatinine, ACC/ASCVD prediction tool, statin use
† Adjusted for gender, body mass index, LDL and creatinine
Copyright American Society of Nuclear Cardiology

## Slide 6
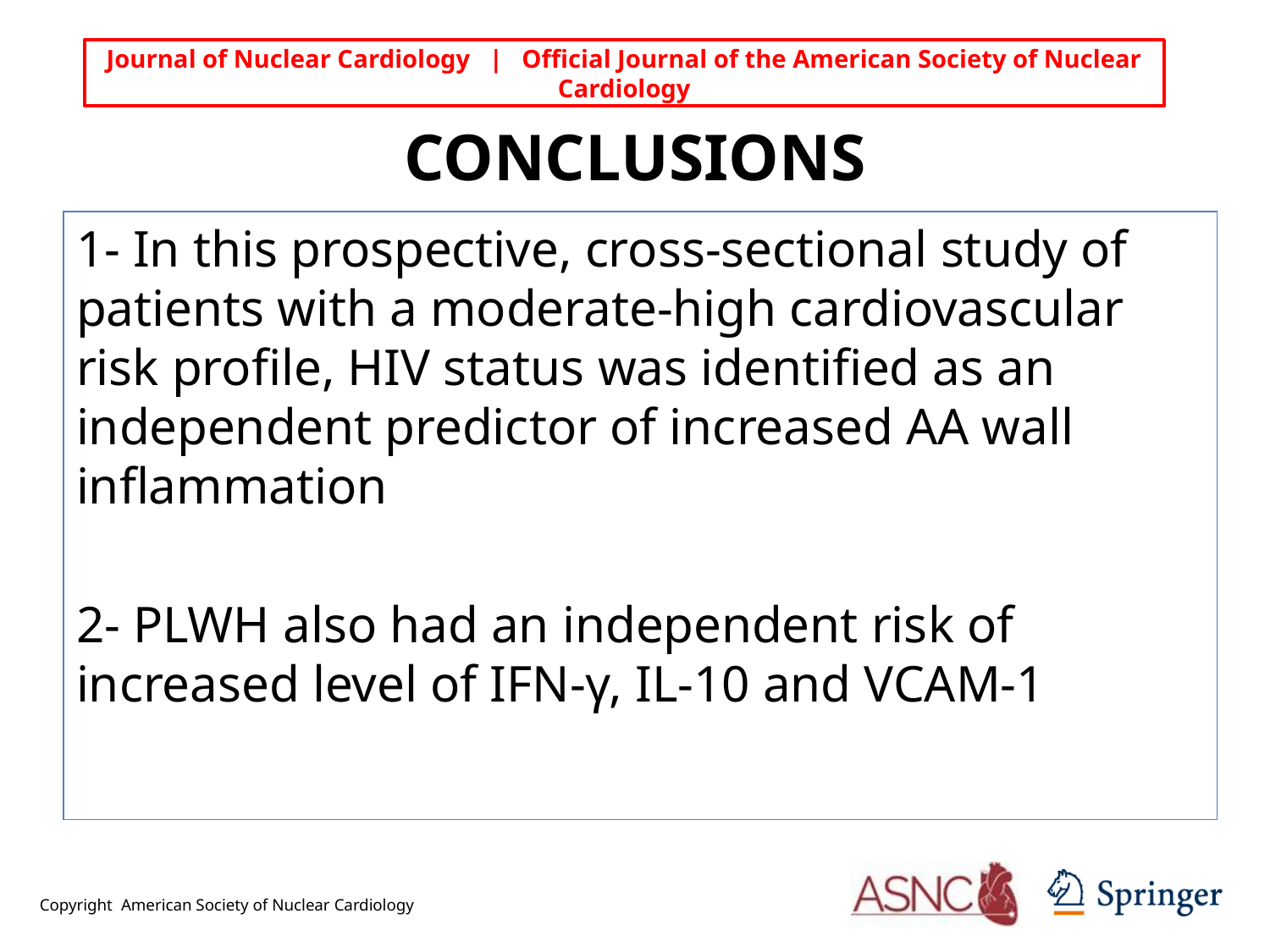

Journal of Nuclear Cardiology | Official Journal of the American Society of Nuclear Cardiology
# CONCLUSIONS
1- In this prospective, cross-sectional study of patients with a moderate-high cardiovascular risk profile, HIV status was identified as an independent predictor of increased AA wall inflammation
2- PLWH also had an independent risk of increased level of IFN-γ, IL-10 and VCAM-1
Copyright American Society of Nuclear Cardiology
